# The Australian Clinical Dosimetry Service: a commentary on the first 18 months

**DOI:** 10.1007/s13246-012-0161-1

**Published:** 2012-09-28

**Authors:** Ivan Williams, John Kenny, Jessica Lye, Joerg Lehmann, Leon Dunn, Tomas Kron

**Affiliations:** 1 Australian Clinical Dosimetry Service, ARPANSA, 619 Lower Plenty Road, Yallambie, VIC 3085 Australia; 2 Peter MacCallum Cancer Centre, Melbourne, VIC 3008 Australia

## Introduction

The Australian Clinical Dosimetry Service, ACDS, is a voluntary national auditing service for radiotherapy centers. Provided free of charge the ACDS is now little over one and a half years old. While an independent audit service has been desired by the radiotherapy professions, (Radiation Oncology, Medical Physics and Radiotherapy) for a few decades, the dosimetry incidents at Adelaide and Coffs Harbor stimulated all levels of government in Australia. While not very significant in comparison to international incidents, they were important in convincing the Government to act. One outcome was a formal agreement for the Australian Department of Health and Ageing (DoHA), to fund a 3 year trial program of an Australia-wide radiotherapy dosimetry audit program. In 2010, the Australian Radiation and Nuclear Safety Agency (ARPANSA), signed a Memorandum of Understanding (MoU) with DoHA to establish the auditing service (ACDS).

This article is intended to be the first of an annual communication from the ACDS to the radiotherapy professions informing them about the methods and rationale behind the ACDS’ actions. This article focuses on the initial setup of the ACDS, the design of the audit structure, discussion points arising from the audits performed so far and our immediate goals. It is also an invitation for comment and discussion about the ACDS procedures and function, and its future.

In February 2011, the Australian government formally launched the Australian Clinical Dosimetry Service, a radiotherapy auditing service unique in its scope, oversight and assessment:

### Scope

Over an initial three year trial period dosimetric audits will be provided free of charge to all public and private radiotherapy providers in Australia. Participation is voluntary. Over the 3 years, the ACDS must develop a three level audit program and capture 80 % of the existing Linacs in Australia and at least 50 % of all new Linac installations.

### Oversight

The MoU defines the three level audit structure, prescribes reporting to DoHA and milestones required for on-going funding. It also mandates and details the formation of a Clinical Advisory Group (CAG). The CAG comprises representatives from the professional organisations in Australia covering Radiation Oncologists, Radiation Therapists and Medical Physicists, private practice, the Radiation Oncology Reform Implementation Committee (RORIC), and the Trans-Tasman Radiation Oncology Group (TROG). The CAG provides expert advice to the ACDS and reviews the audit development and all documentation associated with the audits.

### Trial assessment

In 2013, the final year of the 3 year trial, the ACDS will be reviewed by an independent assessor. At a minimum, the reviewing body will seek information, from centres which the ACDS has audited, State and Federal governments, patient groups and the ACDS itself. After collating and assessing the information, the reviewer will recommend to the Government whether to continue, modify or terminate the ACDS.

### Initial months

The plan described above was first publically presented by the ACDS at the Engineering and Physical Sciences in Medicine conference at the Melbourne Cricket Ground in December 2010. The presentation was well received and led to immediate requests for dosimetry measurements which were more accurate than the existing postal TLD audits previously performed by ARPANSA and adopted by the ACDS. The main drive for this additional service came from the heads of radiotherapy departments who found themselves responsible for some of the new geographically isolated radiotherapy centres being built around Australia. They requested an on-site ionisation chamber measurement, especially for new machines prior to their clinical use. In response to this request the ACDS developed plans for an on-site independent audit. This audit eventually became the Level Ib audit described below. However, all these plans would be moot without the staff to fulfill them.

A recruitment program in late 2010 resulted in the ACDS being in the fortunate position to offer positions to a skilled group of physics professionals with considerable expertise across all aspects of megavoltage dosimetry, clinical practice and computer modelling. Over 2011 John Kenny, Jessica Lye and Joerg Lehmann arrived at the ACDS and the audit planning became audit delivery.

### Audit design

The ACDS was able to review and build on existing international audit programmes. The IAEA/WHO, European Society for Therapeutic Radiology and Oncology, ESTRO, and the Radiological Physics Centre (RPC) in Houston Texas all have published on their comprehensive experience over many decades [1–6]. The ACDS had a distinct ‘second-mover’ advantage, entering a field which was populated with numerous competent examples, including the ARPANSA thermoluminescent detector (TLD) audit, which the ACDS inherited and adopted as its first Level I audit.

**Fig. 1:**
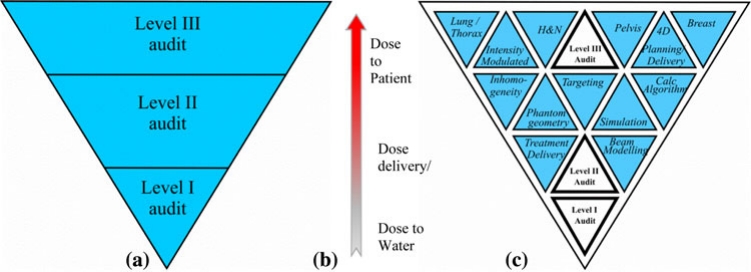
A triangular schematic of the tiered audit system used by the ACDS to demonstrate the inter-dependent nature of the three Level audit service. Reviewing (**a**) in the clinical environment the dose to water, (**b**) obtained at the standards laboratory, needs to be convolved through the treatment complexities of planning systems, delivery techniques and human anatomies etc., (**c**), to accurately determine the dose delivered to the patient in different anatomical sites. The triangular structure reflects the clinical dose delivered to patients, independent of the complexity of the treatment technologies, depends fundamentally upon the basic dosimetry, that the dose delivered by a linear accelerator to water, is correctly performed by the clinic
(ii) Features audits that build on each other thereby allowing for trouble shooting of a higher level audit by the use of a lower level one.
(iii) Always connects the more complex audit levels (II and III) back to Level I by including measurement analogous to a reference conditions.
(iv) Are performed by the staff within a radiotherapy facility who would normally perform the role.
(v) Uses a suite of audits that should enable the reasons for non-compliance to be identified.
(vi) Maximises the similarity between the protocols and reporting process for all audit levels.

The ACDS reviewed the existing audit techniques and built a three level audit structure which:

(i) Progresses from single point dose to water under reference conditions to dose to the patient, as shown in Fig. 1.

As well as the dosimetric requirements, the ACDS has developed audit management and data handling systems. Methods were built from scratch and modified as required with the following precepts:

(i) The audit system should demonstrate the potential for longevity.
(ii) The logistics and management of the audits service would have to be solid and durable—the ACDS had to develop systems that could track multiple overlapping audits for centres around Australia.
(iii) The initial trial period had to develop an audit and data management structure which would be capable of expanding to incorporate new audits.

The three level audit structure adopted by the ACDS was required by the MoU and corresponds to the standard definitions in the literature [7, 8]. All other audits, measurements and treatments are predicated on the Level I audit, i.e. the reference dosimetry, being correct.

The audit results are determined by the percentage deviation of the facility stated dose output from the ACDS determined dose output, for each clinical beam.

### Level I audit

**Fig. 2:**
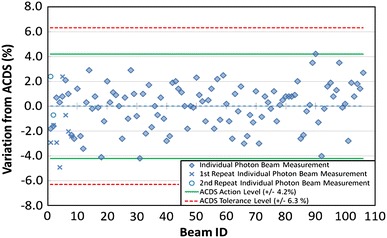
ACDS Level I (TLD) audit outcomes for 57 linacs measurements from its inception to the 7th August 2011. The *dashed line* just above the zero percentage variation is the mean of all readings

The Level I audit is a remotely conducted measurement with a passive dosimeter. The measurement is directly related to reference conditions and thus verifies that the output of the linac is in accordance with the operator’s expectation. Examples include the IAEA TLD audit and RPC’s photon beam optically stimulated luminescent detectors (OSLD) audits. Over the first few months of 2011 the ACDS reviewed existing alternative dosimeter systems and resolved to test the Landauer MicroStar nanoDot OSLD system as an alternative to the powdered TLD system (Landauer, Inc., Glenwood, IL). Testing, Monte Carlo modelling and field trials of the OSLD system resulted in the ACDS moving to it as a remote audit dosimeter on 1st July 2012. As part of the OSLD release the ACDS created instruction videos that have been posted on the internet [9, 10]. At present, the audit is for photon beams only, in 2013 the ACDS intends to extend the Level I OSLD audit to include electron beams. The audit outcomes from 1 January 2011 to 7 August 2012 are shown in Fig. 2. At the time of writing the ACDS has performed Level I audits on more than 50 % of all the lina cs in Australia (Fig. 2).

### Level Ib audit

Designed outside the requirement of the MoU and in response to requests by chief physicists, the Level Ib involves a trained physicist from the ACDS taking reference class chambers to a treatment centre and measuring the beam. It was developed outside the MoU requirements in response to demand from the professional community. Farmer-type and Roos chambers are used in the centre’s water tank, recombination and polarity measurements are taken and the centre is given an ACDS calibration factor for photon and electron beams. The Ib audit has been very popular with requests averaging at about one every 2 months.

### Level II audit

The Level II audit investigates the planning system’s capacity to accurately model more demanding situations. The Level II audit employs a number of successively more complex geometries involving asymmetric and wedged beams, and a lung volume. Measurements are performed at two depths with a two dimensional array. Designed with knowledge of anthropomorphic Level III audit, and IAEA TECDOC 1583 [11], the Level II audit was also intended to be able to deconvolve the more complex Level III audit in the case of an unexpected audit outcome. This will enable the troubleshooting of an unexpected Level III audit outcomes through the Level II irradiations.

Synthetic DICOM files of the audit arrangement are provided to the centre being audited eliminating confounding imaging issues. The arrangement ensures that the planning system is being tested rather than the imaging devices. The audit includes non-reference fields encompassing field asymmetry and wedges with measurements performed at 7 cm and 15 cm water equivalent depths. The most challenging tests of the Level II audit incorporate a lung volume within the solid water above the 2D array. The Level II audit is approaching the end of field testing and should be released for clinical auditing in the last quarter of this year.

### Level III audit

The Level III audit simulates the passage of a patient from imaging to treatment. It is an end-to-end investigation which compares the doses calculated at selected points by a planning system with measurements performed by the ACDS. The centre performs a CT of the computerised imaging reference system (CIRS) anthropomorphic phantom and transmits the DICOM data set to their planning system where the contouring, planning and calculations are performed. The centre then sends the treatment data through the local patient data management system to the linac and perform all the standard quality assurance checks. The ACDS then positions the phantom for treatment and the centre’s staff performs the irradiation while the ACDS measures the output.

The Level III audit fields are drawn from the IAEA TECDOC-1583 [11], and are comparable to clinical fields utilized on patient treatment. In contrast with the Level II audit, the phantom is imaged by the centre being audited. The CT data is sent to the planning system, treatment plans are generated and any standard QA checks are performed. The Level III audit has been clinically released after field trials at four volunteer centres and has been used to formally audit five facilities. The data obtained from these procedures has been divided into two cohorts. The first cohort is the measurements which are obtained from homogenous phantom regions within the primary beam, where;

(i) the ACDS has a high degree of confidence in the measurement,
(ii) it is reasonable to assume that the planning system should be able to calculate the dose with accuracy, and
(iii) the position is of direct clinical interest.

For these measurements, the ACDS bases its audit pass criteria on a 5 % tolerance, in-line with internationally accepted clinical dosimetric variance [12, 13].

The second cohort of data is obtained from measurements outside the direct beam or the in the lung. At this time these points are compared to the planning system calculation and provided to the auditee as a reported not scored, RNS, outcome. The data is collated by the ACDS and will be used to review the expected accuracy of planning systems when calculating dose outside the primary beam path. It is envisioned that the ACDS will report these points in the future.

### Initial recommendations

The ACDS has now performed a large number of on-site and remote audits and, while agreeing that the plural of anecdote is not data, has noticed a few issues which contribute to imperfect audit outcomes.

The first issue relates to barometers. Pressure corrections for dosimetry measurement are linear with measured pressure. A barometer inaccuracy of *x* % produces an immediate dosimetry error of *x* %. The ACDS has found at least three barometers which gave readings up to 1 % different to the calibrated barometers we bring to audits. We have recommended a number of centres have their barometers re-calibrated at a National Australian Testing Agencies (NATA) accredited laboratory where the accreditation certificate explicitly includes barometer calibrations.

The second issue relates to legacy dosimetry procedures. In a number of centres the ACDS has investigated dose anomalies during an audit and found that measurement protocols for routine quality assurance have not been periodically reviewed. While these issues frequently resolve, they have been found to be responsible for local dose inaccuracy between 1 and 2 percent. Although the higher links in the dosimetric chain, the local standard for example, are well maintained and understood with high quality control, this can reduce further away from the local standard. The ACDS has recommended that the protocols establishing the dosimetric chain, often spreadsheets, are reviewed and updated by the physicists who use them. The revised spreadsheet should then be formally adopted and then presented to the rest of the department.

### Closing statement

To-date the ACDS has audited over half the facilities in Australia with at least one audit. In a number of instances, the ACDS has determined that a facility should mitigate a dosimetric issue and has formally recommended a specific action. Analysis of audit outcomes is beginning to indicate correlations between facility practice and audit outcome. It is too early to ascribe causation at this point in time.

The entire ACDS team will be at the Gold Coast for the EPSM2012 conference and we look forward to some interesting discussions. Please give us feedback about this commentary and our audits. It is worth remembering that the ACDS is not permanent, will be independently reviewed in 2013. So if you want an independent dosimetric auditor to continue in the existing format or another, you need to convey that to the reviewer.
